# Urinary CD8+HLA-DR+ T Cell Abundance Non-invasively Predicts Kidney Transplant Rejection

**DOI:** 10.3389/fmed.2022.928516

**Published:** 2022-07-15

**Authors:** Emil Grothgar, Nina Goerlich, Bjoern Samans, Christopher M. Skopnik, Diana Metzke, Jan Klocke, Luka Prskalo, Paul Freund, Leonie Wagner, Michael Duerr, Mareen Matz, Sven Olek, Klemens Budde, Alexander Paliege, Philipp Enghard

**Affiliations:** ^1^Department of Nephrology and Intensive Care, Charité–Universitätsmedizin Berlin, Corporate Member of Freie Universität Berlin and Humboldt-Universität zu Berlin, Berlin, Germany; ^2^German Rheumatism Research Center Berlin (DRFZ), Berlin, Germany; ^3^Berlin Institute of Health (BIH) at Charité–Universitätsmedizin Berlin, Berlin, Germany; ^4^Ivana Türbachova Laboratory for Epigenetics, Precision for Medicine GmbH, Berlin, Germany; ^5^Universitätsklinkum Carl Gustav Carus, Dresden, Germany

**Keywords:** transplantation, kidney, urine, T cell, biomarker, CD8+HLA-DR+, allograft acute rejection, tubular epithelial cell

## Abstract

Early detection of kidney transplant (KT) rejection remains a challenge in patient care. Non-invasive biomarkers hold high potential to detect rejection, adjust immunosuppression, and monitor KT patients. So far, no approach has fully satisfied requirements to innovate routine monitoring of KT patients. In this two-center study we analyzed a total of 380 urine samples. T cells and tubular epithelial cells were quantified in KT patients with graft deterioration using flow cytometry. Epigenetic urine cell quantification was used to confirm flow cytometric results. Moreover, a cohort of KT patients was followed up during the first year after transplantation, tracking cell subsets over time. Abundance of urinary cell counts differed in patients with and without rejection. Most strikingly, various T cell subsets were enriched in patients with T cell-mediated rejection (TCMR) compared to patients without TCMR. Among T cell subsets, CD8+HLA-DR+ T cells were most distinctive (AUC = 0.91, Spec.: 95.9%, Sens.: 76.5%). Epigenetic analysis confirmed T cell and tubular epithelial cell quantities as determined by flow cytometry. Urinary T cell abundance in new KT patients decreased during their first year after transplantation. In conclusion urinary T cells reflect intrarenal inflammation in TCMR. T cell subsets yield high potential to monitor KT patients and detect rejection. Hereby we present a promising biomarker to non-invasively diagnose TCMR.

## Introduction

With a global prevalence of 9–15%, and rising, chronic kidney disease is a major contributor to morbidity and mortality worldwide (1, 2). Kidney transplantation is the therapy of choice in end stage kidney disease (3). However, allograft rejection (AR) leading to reduced allograft function or even graft loss remains a major challenge affecting more than 10 % of patients within the first year after transplantation (4). Established parameters like serum creatinine and proteinuria do not provide definite information about graft pathology and only increase once allograft function is already impaired (5). Transplant biopsy, the diagnostic gold standard to detect rejection, is limited by its invasive nature.

Previous studies discovered that non-invasive biomarkers hold high potential to detect rejection, adjust immunosuppression and monitor kidney transplant (KT) patients (6, 7). Various omics-based urinary biomarkers correlated with kidney inflammation and rejection (8–10). Apart from soluble factors, urine samples serve as non-invasive source for cellular components derived from the allograft. Such urinary cells hold potential as AR biomarkers since they may reflect detrimental processes in the transplant. Our group previously demonstrated that urinary cells can be used to monitor kidney damage and kidney inflammation precisely (11, 12). Other groups linked urine-derived cells to AR (13–15). More specifically, urinary HLA-DR+ cells and CD8+ T cells analyzed by flow cytometry (FC) have been suggested as promising biomarkers to detect rejection (13, 15–18). Previous trials also reported tubular epithelial cells (TEC) to represent damage in AR (19–21). Our group recently developed a biomarker combination involving urinary T cells and TEC detected by FC to identify patients with kidney transplant rejection (22).

However, many of the proposed biomarkers showed insufficient sensitivity and specificity, and were often only analyzed in small and single-centered explorative trials. Accordingly, diagnostic yield of promising biomarkers could not be proven in confirmatory trials if they had been done at all.

The current study extends previous research by (a) validating our previous findings in a multi-center setting, (b) adding an additional method (epigenetic qPCR analysis) proving the concept of urinary cells as non-invasive biomarker of rejection, (c) performing deeper phenotyping of urinary T cells and (d) describing urinary cell population trajectories during the first year after kidney transplantation to determine biomarker applicability.

This unique design allowed us to comprehensively investigate urinary cells as biomarkers in KT monitoring. To find the putatively best biomarker among T cell subsets, we investigated CD4+, CD8+, effector memory, central memory, effector memory T cells re-expressing CD45RA (termed TEMRA), and HLA-DR+ T cells. Additionally, as a surrogate for intrarenal tissue damage urinary proximal and distal TEC were quantified.

## Methods

### Patients

**Figure 1:**
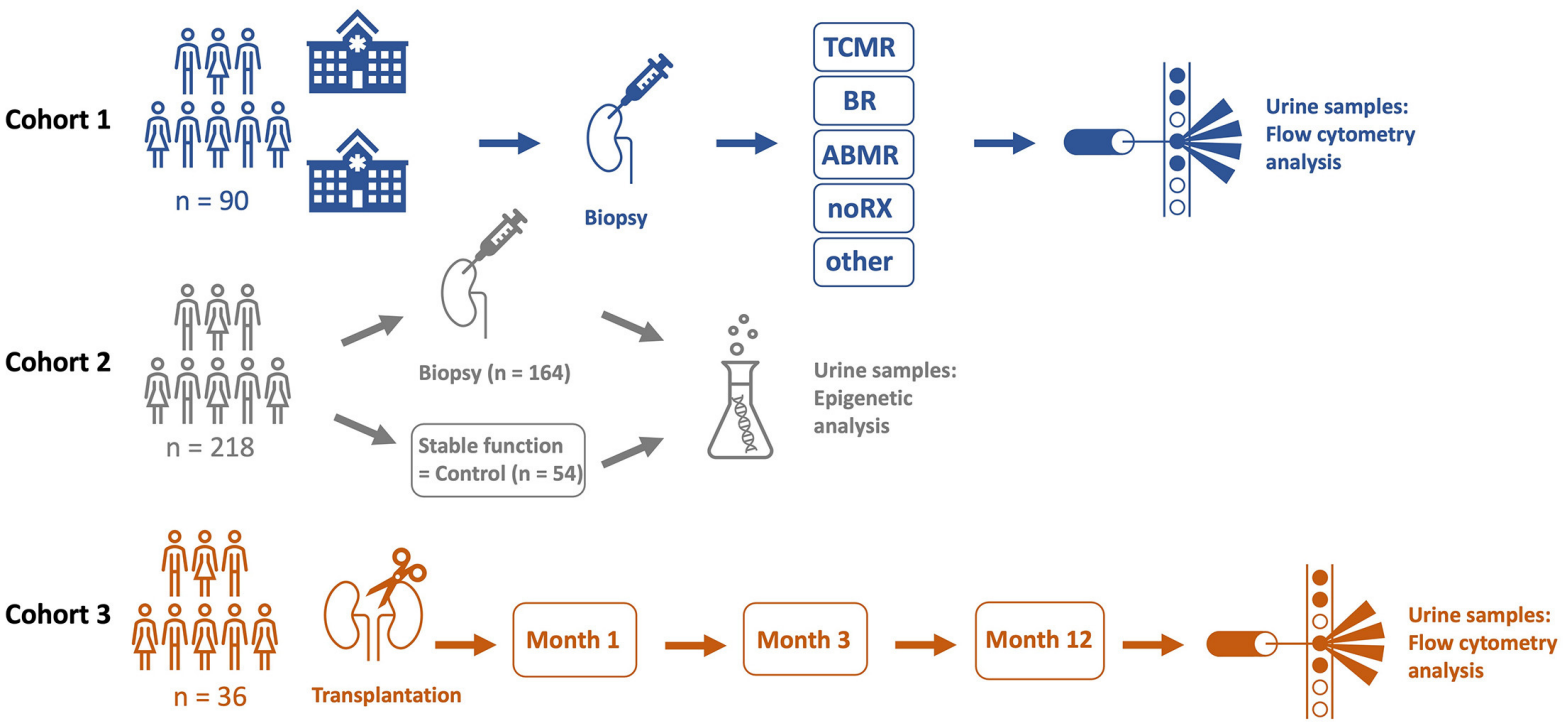
A total of three different cohorts were analyzed in this trial. Cohort 1 included 90 kidney transplant (KT) patients from two hospitals (Charité University Hospital, Berlin, Germany and Carl Gustav Carus University Hospital, Dresden, Germany) who underwent kidney biopsy due to graft deterioration. Patients were categorized by histopathological diagnosis and urine samples were analyzed by flow cytometry. In cohort 2, urine samples of 218 KT patients were subject to epigenetic qPCR analysis. 164 patients of cohort 2 underwent kidney biopsy because of graft deterioration, 54 stable KT patients served as a control group. Cohort 3 included 36 KT patients. Urine samples were analyzed on three scheduled visits by flow cytometry in a follow-up setting during the first year after transplantation.

380 urine samples of KT patients were analyzed in three different cohorts. Detailed patient characteristics are shown in Table 1, schematic illustration of cohorts is presented in Figure 1.

**Table 1:** Patient characteristics.

**Characteristic**	**Cohort 1**	**Cohort 2**	**Cohort 3**
Mean age in years ± SD	55 (± 14)	51 (± 16)	54 (± 13)
Male/Female	54/36	100/41	19/17
Mean years post KT ± SD	6 (± 7)	5 (± 6)	First year follow-up
**KT donor**			
Living related	20	21	6
Living unrelated	13	28	5
Cadaveric	57	92	25

*Demographic details of patients included in statistical analysis. Patients who failed quality control for epigenetic analysis are not show*.

For cohort 1, we collected 90 urine samples between 2019 and 2021 for flow cytometric analysis from patients with graft deterioration and diagnostic biopsy of the Department of Nephrology, Charité University Hospital, Berlin and from Carl Gustav Carus University Hospital, Dresden, Germany.

For cohort 2, between 2010 and 2018, 218 urine samples were collected from patients at the Department of Nephrology, Charité University Hospital, Berlin and were subject to epigenetic analysis. Among these samples, 164 were collected from patients with graft deterioration and, as control group, 54 from patients with stable graft function, defined as no fluctuation of more than +/– 0.3 mg/dl creatinine compared to the prior visit. Professional diagnoses by board certified nephropathologists from renal biopsies served to uniquely group graft deterioration into borderline rejection (BR), T cell mediated rejection (TCMR), and antibody mediated rejection (ABMR), other specific pathohistological diagnosis (other), or no rejection (noRX). Children, patients on menstruation, patients with overt causes for transplant deterioration other than rejection, such as urinary tract infections or postrenal causes of acute kidney injury, and patients with already commenced rejection therapy were excluded from the study.

For cohort 3, 72 samples from newly transplanted patients were collected as follow-up during the first year after transplantation. Differences in urinary cell trajectories during that period may prospectively identify patients developing rejection. Planned urine sample acquisitions at one, 3 and 12 months after transplantation were subject to variation in schedule due to the COVID-19 pandemic. Sample collection was done at the Department of Nephrology, Charité University Hospital, Berlin.

### Sample Preparation

For cohort 1 and 2, we collected urine samples up to 72 h prior to transplant biopsy. Samples from prospective cohort (cohort 3) were collected on scheduled follow-up visits. We used spontaneously voided urine. We developed a urine-cup-based fixation system with imidazolidinyl urea (IU, Sigma-Aldrich) and 3-(N-morpholino)propanesulfonic acid (MOPS, Carl Roth GmbH + Co. KG) to preserve urine samples (23). Specimen were stored at 4°C for up to 7 days, centrifuged (600 g, 6 min) and frozen in 90% fetal calve serum (FCS) and 10% dimethylsulfoxide (DMSO) (cohort 1 and 3). Preparing samples for epigenetic qPCR analysis (cohort 2), urine specimen was centrifuged immediately (1,500 g, 10 min) and frozen at −80°C. All samples were stored at −80°C for a median of 3 years.

**Figure 2:**
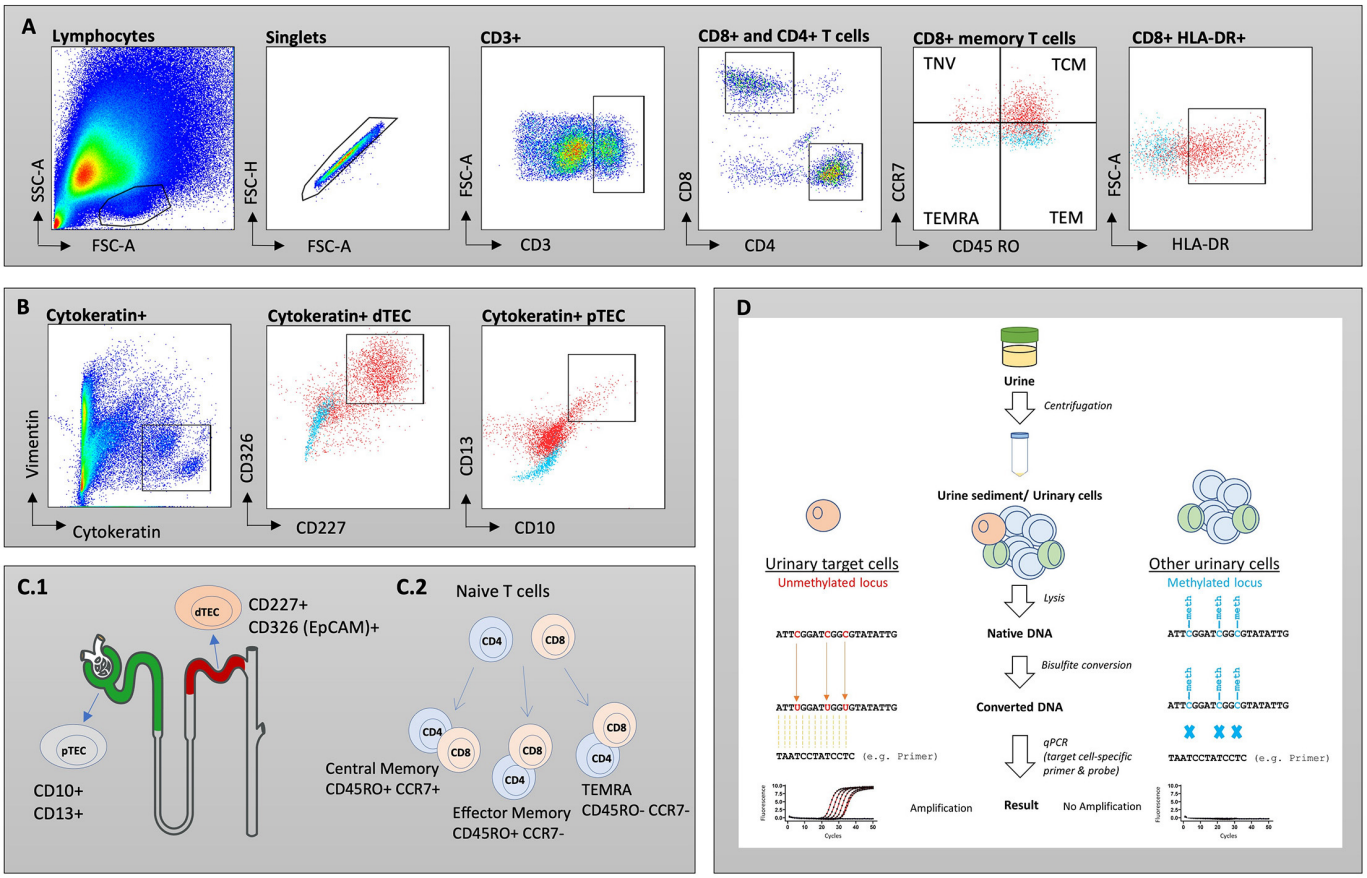
Gating strategies for T cell subsets **(A)** and tubular epithelial cells (TEC) **(B)**. Isotype controls are displayed as blue, while full stains are represented in red. **(C.1)** Schematic overview of investigated subsets. Proximal TECs were defined CD10+ and CD13+, while distal TECs were characterized being CD227+ and CD326(EpCAM)+. **(C.2)** Maturation of naïve T cells into memory T cells. **(D)** Workflow for epigenetic analysis of urine samples. SSC, side scatter; FSC, forward scatter; TNV, naïve T cells; TEM, T effector memory cells; TCM, T central memory cells; TEMRA, T effector memory cells re-expressing CD45RA.

To conduct flow cytometry analysis, we defrosted samples in phosphate-buffered saline (PBS), pH 7.2 with 0.2 % bovine serum albumin (BSA) and 2 mM Ethylenediaminetetraacetic acid (EDTA) (PBE) and strained through a 30 μm cell strainer (Miltenyi Biotech). *PermWash 10X Solution* (BD) was used to permeabilize cells for intracellular staining of TEC. Fc receptors were blocked with *FcR Blocking Reagent (human)* (Miltenyi Biotech) to reduce unspecific binding and labeled for 15 min on ice with fluorochrome-conjugated monoclonal antibodies in the dark. The following antibodies were used: for T cells anti-CD3-APCeF780 (eBioscience, SK7, mo IgG1k), -CD4-PEVio770 (Miltenyi Biotec, REA623, REA) -CD8-APC (Biolegend, SK1, mo IgG1k) -CD45RO-PE (Biolegend, UCHL1, mo IgG1k2), -CD45-BUV805 (BD, 3D12, rat IgG1ak), -CCR7-BV421 (Biolegend, G043H7, mo IgG2ak), -HLA-DR-BUV395 (BD, G46-6, mo IgG2ak), -CD28-FITC (Biolegend, CD28.2, mo IgG1k) and for tubular epithelial cells anti-Cytokeratin-FITC (Miltenyi Biotec, CK3-6H5, mo IgG1k), -Vimentin-APC (Miltenyi Biotec, REA409, REA), -CD10-PeVio770 (Miltenyi Biotec, REA877, REA), -CD13-APCVio770 (Miltenyi Biotec, REA263, REA), -CD227-PE (Miltenyi Biotec, REA448, REA), -CD326-BV711 (Biolegend, 9C4, mo IgG2b). Samples were analyzed on a BD FACSymphony™ A5 Cell Analyzer. Gating strategies are depicted in Figures 2A,B. Acquired cell numbers were normalized to a volume of 100 mL urine. FC data was analyzed with *FlowJo 10.7* (BD Biosciences).

For epigenetic analysis, DNA from urine was obtained, processed, and analyzed using the method published by *Pradhan et al*. with some modifications (24). Workflow for epigenetic analysis of urine samples is depicted in Figure 2D. In short, urine sediment (~75 μl) was lysed by adding 67 μl lysis buffer [54.25 μl ATL buffer (Qiagen), 9 μl Proteinase K (30 mg/ml, CAS 39450-01-6)], and 3.75 μl spiking plasmid essential for absolute quantification (400.000 copies/μl, Genscript) to urine sediment followed by an incubation step (56°C for 1.5 h, 900 rpm) to make genomic DNA of urinary nucleated cells accessible for bisulfite-treatment. Bisulfite-conversion was performed by adding 270 μl ammonium bisulfite [65–75% (w/w), CAS-No.: 10192-30-0] and 90 μL of tetrahydrofurfuryl alcohol (THFA, purity ≥ 98%, CAS No.: 97-99-4). After bead-based purification (Dynabeads My Silane Genomic DNA Kit, Invitrogen), a qPCR-based approach (demethyl-specific primers and probes) was used to determine CD3+ and CD3+CD8+ T cells and proximal TEC based on cell type-specific demethylated genomic regions. Cell type-specific epigenetic markers were identified by bisulfite-sequencing and cell counts were calculated according to Baron et al. (25) (Supplementary Figure 1). Oligonucleotides for bisulfite-sequencing and for demethyl-specific qPCR are listed in Supplementary Table 1.

### Statistical Analysis

Mann-Whitney test was used to test for significantly different cell counts between groups with *p* < 0.05 being considered as significant. Friedman and Wilcoxon test were used to detect differences in the longitudinal cohort. Bonferroni correction was used to correct for multiple testing. Medians, means, Mann-Whitney, Friedman, and Wilcoxon tests, Bonferroni correction and receiver operating characteristic (ROC) curves were calculated using R version 4.1.0. (26).

## Results

### Urinary T Cell Abundance Is Enriched in TCMR

**Figure 3:**
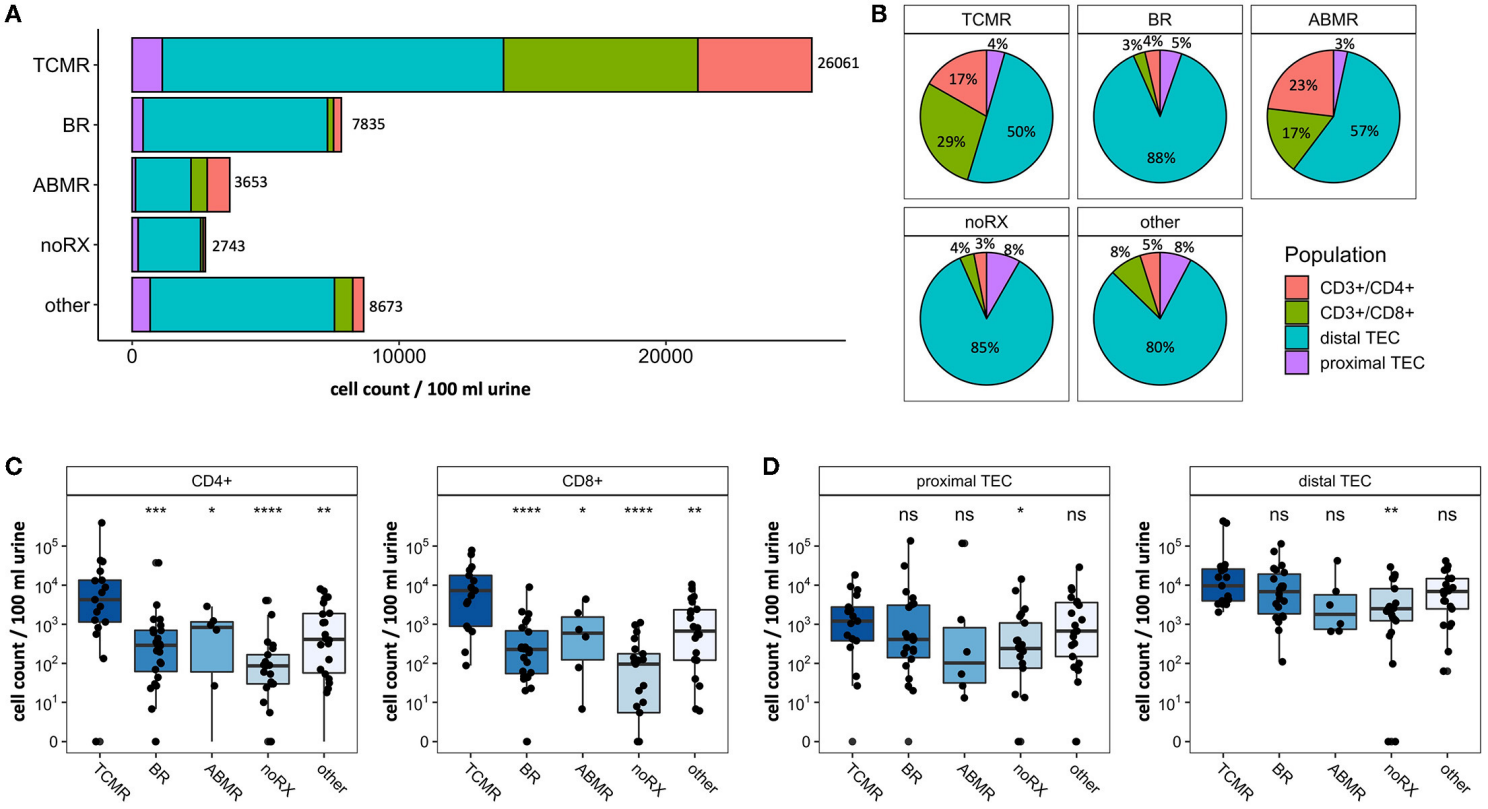
Absolute cell counts recorded by flow cytometry in patients undergoing renal biopsy due to graft deterioration. Patients are subdivided into five groups based on histopathological results from biopsy. **(A)** Stack plot for population proportions. Each stack illustrates the mean absolute cell count per population in each group. **(B)** Pie charts representing composition of urinary cells (selected populations) per group. **(C)** CD4+ and CD8+ T cell counts per 100 ml urine shown for different biopsy groups. **(D)** Proximal and distal TEC counts per 100 ml urine shown for different biopsy groups. Significance levels indicate comparison with TCMR; ns, no significance; *p < 0.05; **p < 0.01; ***p < 0.001; ****p < 0.0001. TCMR, T cell-mediated rejection; BR, Borderline rejection; ABMR, antibody-mediated rejection; noRX, no rejection; other, other pathologies; TEC, tubular epithelial cell.

To study populations of T cells and TEC derived from urine in patients with kidney graft deterioration, we grouped participants based on the results of their KT biopsy. In cohort 1, 17 patients were diagnosed with TCMR, 24 patients with BR, 6 patients showed ABMR, 21 patients were grouped as noRX and 22 patients presented with other specific pathologies on their biopsy results. All 90 urine samples of this cohort were analyzed by FC. Patients with inconclusive biopsy results were excluded from statistical analysis. Stack plots shown in Figure 3A give an overview of cell counts per population in each group. Patients with TCMR presented with the most urinary cells in total (26,061 cells/100 ml urine on average). Together with ABMR patients, they also had the highest fraction of urinary immune cells (combined CD4+ and CD8+ fraction: 40–46%, Figure 3B). In contrast, patients with BR, noRX or other graft pathologies presented predominantly with distal TEC (Fraction: 80–88%, Figure 3B). The fewest urinary cells were found in patients with noRX (2,743 cells/100 ml urine on average). Patients with TCMR presented with significantly increased urinary CD8+ T cell counts per 100 ml urine compared to patients with other biopsy results (TCMR vs. BR: *p* < 0.0001; TCMR vs. ABMR: *p* < 0.05; TCMR vs. noRX: *p* < 0.0001, TCMR vs. other: *p* < 0.01). CD4+ T cells showed a likewise tendency (TCMR vs. BR: *p* < 0.0001; TCMR vs. ABMR: *p* < 0.05; TCMR vs. noRX: *p* < 0.0001, TCMR vs. other: *p* < 0.001; Figure 3C).

In addition to T cells, we quantified subsets of urinary TEC (Figure 3D). Schematic overview of analyzed TEC populations is depicted in Figure 2C.1. Proximal TEC, defined as Cytokeratin+, CD10+ and CD13+, did not differ significantly between patient groups. In contrast, cell counts of distal TEC (Cytokeratin+, CD227+, CD326+) were higher in patients with TCMR than in patients with noRX (*p* < 0.05). The ratio of T cells and TEC did not improve discrimination between groups.

### Epigenetic Analyses Qualitatively Confirm T Cell and TEC Quantities as Determined by Flow Cytometry

**Figure 4:**
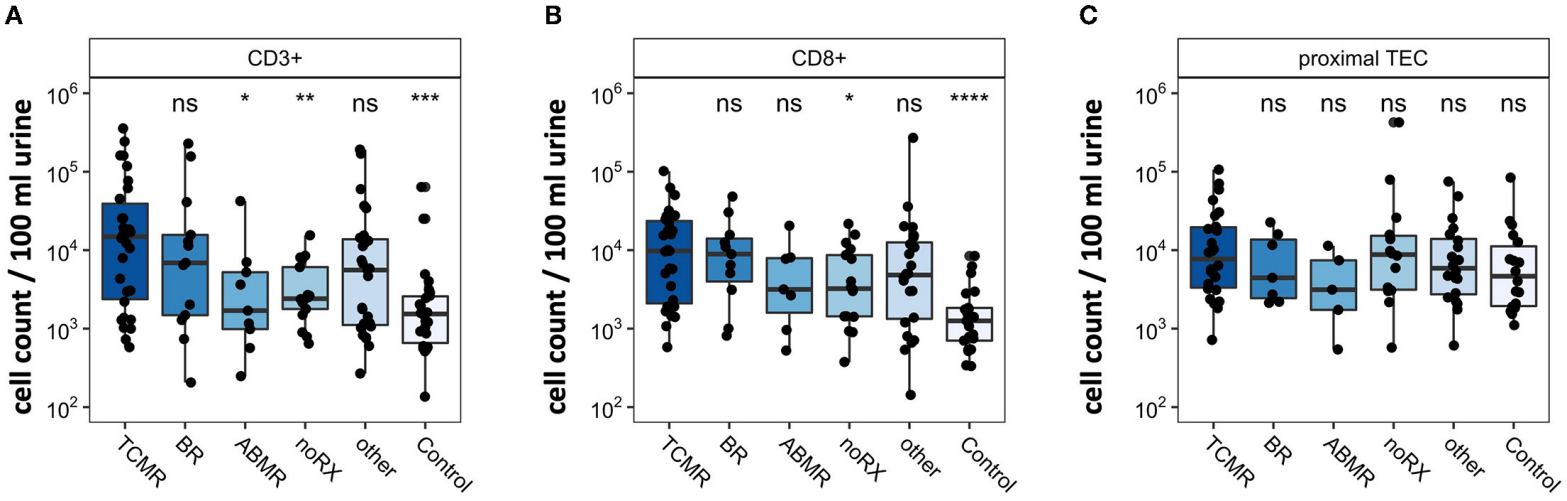
Epigenetic quantification of cell populations in patients with renal biopsy due to graft deterioration. Patients are subdivided into five groups based on histopathological results from biopsy. The sixth group, Control, includes transplant patients with stable graft function. Counts per 100 ml urine were analyzed in **(A)** CD3+ T cells, **(B)** CD8+T cells, and **(C)** proximal TEC. Significance levels indicate comparison with TCMR; ns, no significance; *p < 0.05; **p < 0.01; ***p < 0.001; ****p < 0.0001. TCMR, T cell-mediated rejection; BR, Borderline rejection; ABMR, antibody-mediated rejection; noRX, no rejection; other, other pathologies; TEC, tubular epithelial cell.

For validation purposes, we assessed urinary cells by epigenetic qPCR analysis. In 218 urine samples from kidney transplant patients, we quantified T cells and TEC. The cohort consisted of 164 KT patients with graft deterioration and suspected rejection undergoing transplant biopsy and 54 KT patients with stable kidney function without biopsy as control group. Patients undergoing biopsy were grouped based on histological results. One hundred forty-one samples passed quality control for epigenetic qPCR analysis. They were included in statistical analysis and are depicted in Figure 4. Patients with TCMR showed significantly more CD3+ T cells and CD8+ T cells than patients with noRX or than the control group. Quantity of CD3+ or CD8+ T cells did not discriminate between patients with TCMR and patients with BR or other diagnoses. Epigenetic quantification of proximal TEC showed no difference between disease groups. Therefore, epigenetic qPCR analyses confirmed FC findings showing significantly different amounts of urinary T cells in TCMR, with however imperfect delineation from other patients.

### Subsets of Urinary CD8+ T Cells Enable Improved Discrimination of TCMR

**Figure 5:**
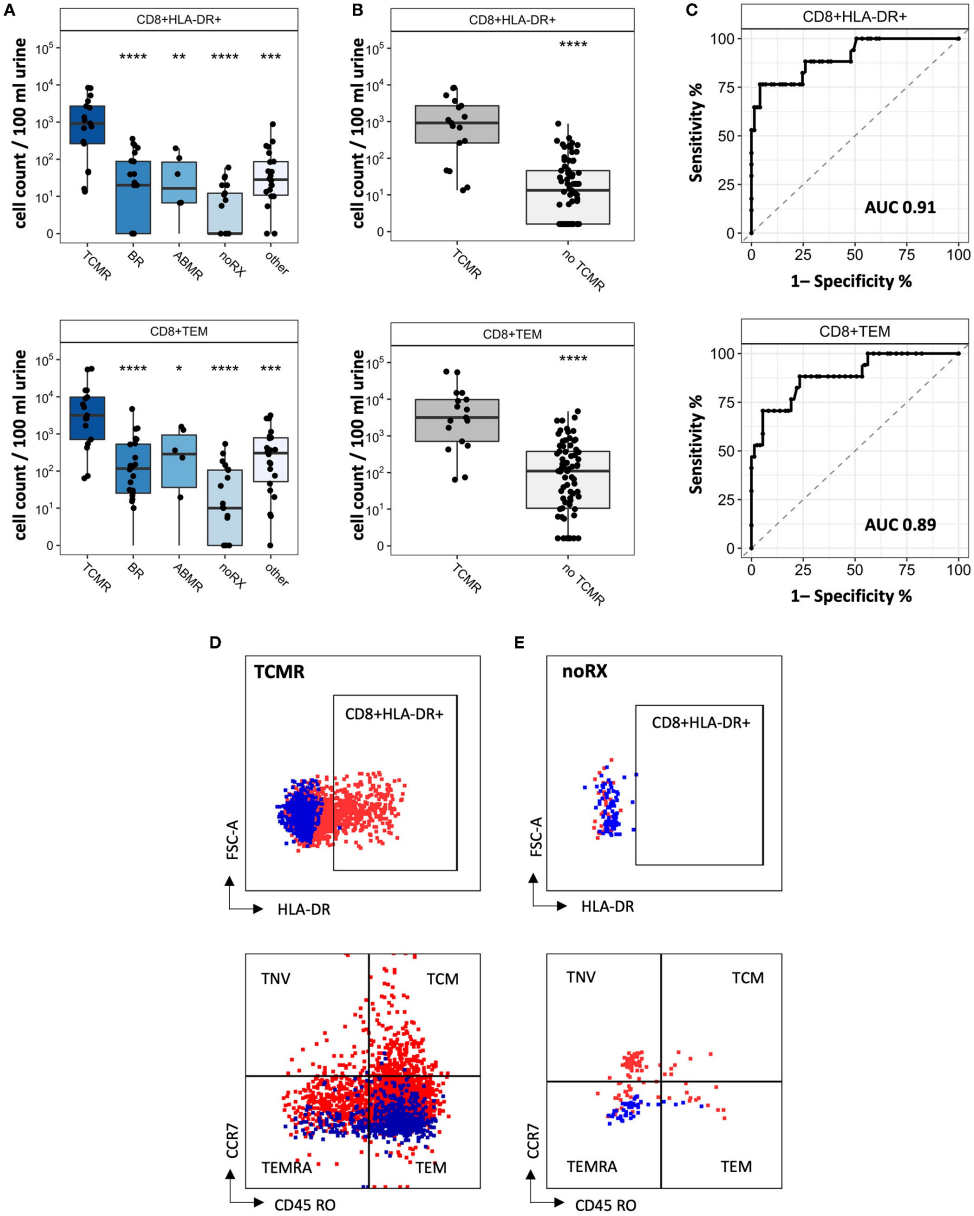
CD8+ T cell subsets as biomarker for detection of KT rejection. **(A)** Cell counts for CD8+HLA-DR+ and CD8+TEM per biopsy group. **(B)** Cell counts from patients with TCMR compared to all other patients (= no TCMR). **(C)** ROC curves to distinguish TCMR from no TCMR. Representative FC gating for CD8+HLA-DR+ and CD8+TEM in **(D)** TCMR patients and **(E)** noRX patients. Isotype controls are displayed as blue, while full stains are represented in red. Significance levels indicate comparison with TCMR; ns, no significance; *P < 0.05; **P < 0.01; ***P < 0.001; ****P < 0.0001. TCMR, T cell-mediated rejection; BR, Borderline rejection; ABMR, antibody-mediated rejection; noRX, no rejection; other, other pathologies; no TCMR, no T cell-mediated rejection; AUC, area under the curve; TNV, naïve T cells; TEM, T effector memory cells; TCM, T central memory cells; TEMRA, T effector memory cells re-expressing CD45RA.

Since CD8+ T cell populations derived from urine showed significant differences in patients with TCMR and patients with other causes of graft deterioration, we further investigated their subsets and activation to optimize their potential as biomarkers to detect rejection. Subsets were quantified for naïve, TEMRA effector memory and center memory T cells. Schematic overview of T cell subsets is depicted in Figure 2C.2. Moreover, HLA-DR+ and CD28+ expression as activation marker was analyzed (Supplementary Figure 2). Most strikingly among CD8+ T cells were CD8+HLA-DR+ and CD8+CD45RO+CCR7- (T effector memory cell, TEM) (Figure 5A, representative gating strategy including isotype controls: Figures 5D,E). Next, we assessed if our analyzed CD8+ subsets were able to distinguish patients with TCMR from all patients without TCMR and found a significant separation between these two groups (noTCMR = BR + ABMR + noRX + others; *n* = 73, TCMR vs. no TCMR: *p* < 0.0001; Figure 5B). To assess the diagnostic ability of CD8+HLA-DR+ and CD8+CD45RO+CCR7-, we calculated ROC curves (displayed in Figure 5C). The area under the curve (AUC) to diagnose TCMR using CD8+TEM cells was 0.89. CD8+HLA-DR+ T cells yielded an even better AUC value of 0.91, resulting in the most promising biomarker to distinguish patients with TCMR from all other patients. Setting a cut-off of 262.5 CD8+HLA-DR+ T cells/100 ml urine shows a sensitivity of 76.47 % and a specificity of 95.89 % to diagnose TCMR.

### Urinary T Cell and TEC Abundance Remain Low Over Time in the First Year After Kidney Transplantation

The first year after kidney transplantation is characterized by a particular high risk for rejection. The intrarenal reorganizing and adaptation processes in that time period after KT may however affect the applicability of biomarkers to detect rejection. In order to assess the applicability of our biomarkers in that time period, we analyzed urine samples of 36 newly transplanted patients. Our goal was to analyze three samples per patient, obtained one, 3 and 12 months after transplantation. Due to COVID19 regulations, clinic visits were canceled or changed to telemedicine visits, resulting in 9 patients each donating only one sample, while 18 other patients only provided two samples during the first year after transplantation. Nine patients fulfilled the initially planned regime of three visits including sample collections (cell trajectories for each individual patient are depicted in Supplementary Figure 3). Only two biopsy proven rejections occurred, diagnosed 3 and 4 months after the last visit and urine analysis in this trial. Therefore, no meaningful comparison of urinary cell counts and rejection was possible.

**Figure 6:**
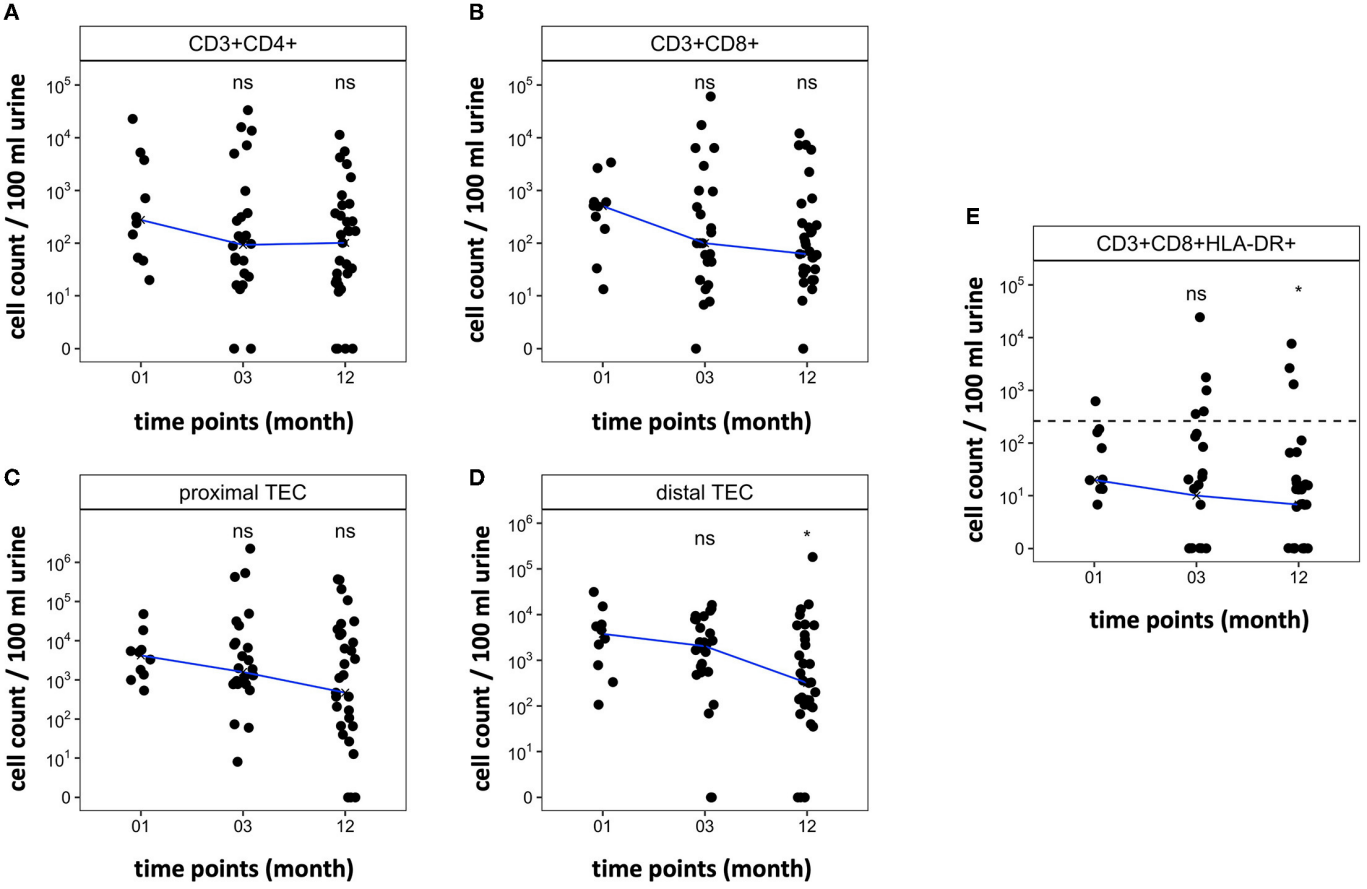
Trajectory of cell counts in patients within the first year after kidney transplantation without rejection. Samples were collected 1, 3, and 12 months post-surgery. Median cell count per time point displayed as line. Cell abundance at different time points was compared. **(A,B)** CD4+ and CD8+ T cell counts decrease within the first year after transplantation. **(C,D)** Cell counts of TEC decrease during the first year after transplantation. **(E)** CD8+ HLA-DR+ T cell populations decrease during the first year after transplantation. Dashed line marks CD8+HLA-DR+ T cell cut-off at 262.5 CD8+HLA-DR+/100 ml urine that showed a sensitivity of 76.47% and specificity of 95.89% to diagnose TCMR in cohort 1. *P < 0.05, ns, no significance; TEC, tubular epithelial cell.

All included patients showed sufficient graft function 12 months after transplantation (creatinine mean 1.77 mg/dl, range 0.9–4.05 mg/dl). Figures 6A–D shows the trajectory of cell counts for CD4+ T cells, CD8+ T cells, proximal TEC, and distal TEC within the first year post transplantation. T cell counts in stable KT patients were low after first month post transplantation (median CD4+: 277 cells/100 ml urine; median CD8+: 506 cells/100 ml urine) and even showed a tendency to decrease over the first year after KT. The trajectories provide insights into regular development of urinary cell counts in patients without complications (defined as biopsy proven rejection, surgical complications or transplant associated hospitalization). Figure 6E shows progression of urinary CD8+ HLA-DR+ T cell populations. Applying our prior calculated cut-off for diagnosing TCMR (line), median cell counts were below cut-off level already 1 month after transplantation. These results suggest that our urine FC biomarker can feasibly be used within the first year after transplantation.

## Discussion

In this first multicenter study on FC urine analysis in KT patients, we reveal CD8+ HLA-DR+ T cells as a potential TCMR biomarker with high precision. Urine FC findings were validated *via* epigenetic analysis and longitudinal analysis of urinary cell abundance over the first year after KT suggest that the biomarker can be applied even in this early, AR-prone phase.

### The Amount of Urinary T Cells Differs Significantly in Patients With and Without TCMR

Urinary T cell counts are significantly increased in TCMR. Our findings regarding CD8+ T cells distinguishing TCMR from other groups are consistent with results of other prior studies (16–18, 22). Abundance of T cells derived from urine even correlated with histopathological findings like tubulitis and interstitial inflammation. This underlines their ability to mirror graft pathology (22). In line with previous research, our findings emphasize the crucial role of CD8+ T cells in rejection. However, while the vast majority of past studies analyzed very small samples sizes, we propose our findings to be more robust due to a larger patient group with rejection and a multicenter setting.

Urinary TEC are abundant in all patient groups with graft deterioration. Contrary to our initial beliefs, we could not show differences in patients with rejection and without rejection, except for significantly more distal TEC in TCMR compared to noRX. The reason for that might be TEC reflecting unspecific kidney damage irrespective of the cause. Additionally, urinary TEC may also reflect increased turnover of the renal epithelium.

### Epigenetic qPCR Analyses Qualitatively Confirmed T Cell and TEC Quantities as Determined by Flow Cytometry

As predicted and assessed by FC, we found higher T cell populations in patients with TCMR using epigenetic qPCR. These findings are in line with abundant previous research stressing T cells' potential as diagnostic tool (13, 16, 17). Epigenetic analysis has been utilized in KT biomarker development in regard to donor-derived cell-free DNA analysis before (27). However, to our knowledge it has not been adapted to analyze urinary cell populations in AR, making this the first trial to apply epigenetic qPCR analysis of urinary cells in patients with graft deterioration. The epigenetic qPCR is an established method for quantifying immune cells in blood or tissues and was used in different studies before (28, 29). Here, this method was applied in addition to FC to validate our findings with a complementary method. Epigenetic qPCR enabled us to analyze samples frozen without any additives stabilizing the cellular integrity as a prerequisite for FC. Using epigenetic qPCR we were able to confirm significantly higher median T cell counts in the TCMR group compared to noRX or Control group in an independent cohort. Due to its methodical robustness, epigenetic qPCR could be an alternative to FC in samples stored without a dedicated protocol for flow cytometric analysis of intact cells.

### Subsets of Urinary CD8+ T Cells Enable Improved Discrimination of TCMR

We found activated CD8+ TEM and CD8+ HLA-DR+ T cell subsets to separate patients with TCMR best from all other examined groups. Pathophysiologically, this makes a lot of sense, since these subsets are suspected to drive tubulitis and interstitial inflammation in AR. Our findings are also in line with previous research, describing HLA-DR positive cells in urine samples with AR (13, 15, 16). With CD8+ HLA-DR+ T cell counts as TCMR biomarker, we surpassed the diagnostic ability of our previously proposed FC TCMR biomarker (22). CD8+ HLA-DR+ cells also show a better performance than transcriptomics and sophisticated urinary protein analyses (9). We think, an implementation of specific urinary cell populations, such as CD8+ HLA-DR+ T cells, to other combined biomarker types, such as *Q Score/Qsant*, could provide powerful precision to diagnose AR (10). However, detection of patients with ABMR *via* FC remains challenging.

### Long-Term Follow-Up of KT Patients Shows Low Amounts of Urinary T Cells and TEC in the First Year in Patients Without Rejection

When examining trajectories of urinary cells within the first year after transplantation, we discovered, as predicted, only moderate urinary cell counts which showed a tendency to decrease over time in patients without rejection episodes. Existing trials assessing prediction of rejection episodes by urine analysis in follow-up settings focus on gross proteinuria (30, 31) or on specific immune cell associated metabolites (32, 33). Our study therefore extends previous findings, shifting its focus on cell populations and their trajectories, which have not been described in a longitudinal setting before. Plus, our results show that cut-off levels for CD8+ HLA-DR+ T cells to diagnose rejection can be applied within the first months after transplantation.

### Practical Implications

Although further studies are needed to draw definitive conclusions, results of our trial present evidence that detailed phenotyping of urinary immune cells with FC provides a promising approach to monitor KT patients and detect rejection. With CD8+ HLA-DR+ T cells revealing the best performance in diagnosing TCMR and the broad availability of FC in routine laboratories, an implementation into clinical care could be realized using existing infrastructure. As suggested by 1 year-trajectories, our biomarker could also be applied within the first year after transplantation and add value in monitoring KT patients.

### Limitations

First, although we conducted a multicentric approach to assess diagnostic performance of urine FC, sample sizes are still confined and rejection incidence (fortunately) is relatively low, making a final evaluation of the diagnostic quality challenging. However, we were able to include patients from two different centers and achieve promising distinction of patients with TCMR from others using FC. Future experimental studies are needed to fully uncover the diagnostic ability of T cell subsets. Second, predictive utility of our non-invasive biomarker candidates remains inconclusive due to low rejection prevalence within the first year in our cohort. Nevertheless, we were able to describe cell population trajectories and share insights into processes within the first year after transplantation. We propose a multicentric longitudinal prospective trial including KT patients to analyze urine samples by FC at regular clinic visits for a longer time span. Lastly, urine FC comes along with certain challenges, such as autofluorescence and issues in investigating rare cell subsets. Therefore, an even deeper phenotyping of immune cells with FC seems effortful. To gain deeper insights, other methods such as mass cytometry or single cell sequencing could provide a solution. More studies are needed to achieve a more fine-grained understanding of “urine prints” among KT patients with graft deterioration. These disease-specific cell patterns might mirror intrarenal pathologies and provide innovative diagnostic tools.

## Conclusion

The current study is a unique investigation phenotyping urinary immune cells by FC as a biomarker to detect KT rejection. We extend previous research by examining urinary cell populations in a multicenter setting and by validating findings conducting epigenetic qPCR analysis. Moreover, this trial includes a longitudinal design to determine biomarker applicability during the most prone timespan for rejection—the first year after transplantation. Our data shows that urinary CD8+ HLA-DR+ T cell have the highest potential to diagnose TCMR, with a cut-off that can be implemented during the first year after transplantation. This study lays the foundation and might catalyze future research exploring urinary immune cell signatures to non-invasively diagnose rejection and monitor KT patients.

## Data Availability Statement

The raw data supporting the conclusions of this article will be made available by the authors, without undue reservation.

## Ethics Statement

The studies involving human participants were reviewed and approved by Charité EA1/284/19. The patients/participants provided their written informed consent to participate in this study.

## Author Contributions

EG and NG conducted flow cytometry experiments and created the manuscript. BS and SO established protocols for epigenetic analysis of urinary cells. BS conducted all epigenetic analyses. CS, PE, and JK established the staining protocol and reviewed the article. DM, LW, PF, and LP provided material and expertise in method development and reviewed the manuscript. MD and KB significantly supported patient recruitment and trial management. MM collected, stored, and processed all samples for epigenetic analysis. PE and AP conceptualized and designed this trial. PE provided intellectual content of critical importance to the work described and gave final approval of the version to be published. All authors supported manuscript writing and gave final approval of the manuscript.

## Funding

This work was supported by grants from the Berlin Institute of Health (BIH) and SPARK Berlin. JK was supported by a research scholarship of the Deutsche Gesellschaft für Nephrologie (DGfN). NG was participant in the BIH Charité Junior Clinician Scientist Program funded by the Charité–Universitätsmedizin Berlin and the Berlin Institute of Health at Charité (BIH).

## Conflict of Interest

BS and SO are employed by Precision for Medicine GmbH. The remaining authors declare that the research was conducted in the absence of any commercial or financial relationships that could be construed as a potential conflict of interest.

## Publisher's Note

All claims expressed in this article are solely those of the authors and do not necessarily represent those of their affiliated organizations, or those of the publisher, the editors and the reviewers. Any product that may be evaluated in this article, or claim that may be made by its manufacturer, is not guaranteed or endorsed by the publisher.

## Abbreviations

ABMR: antibody mediated rejection
AR: allograft rejection
AUC: area under the curve
BR: borderline rejection
BSA: bovine serum albumin
DMSO: dimethylsulfoxide
EDTA: Ethylenediaminetetraacetic acid
FC: flow cytometry
FCS: fetal calve serum
FSC: forward scatter
IU: imidazolidinyl urea
KT: kidney transplant
MOPS: 3-(N-morpholino)propanesulfonic acid
noRX: no rejection
PBE: bovine serum albumin and 2 mM Ethylenediaminetetraacetic acid
PBS: phosphate-buffered saline
ROC: receiver operating characteristic
SSC: side scatter
TCM: central memory T cell
TCMR: T cell-mediated rejection
TEC: tubular epithelial cell
TEM: effector memory T cell
TEMRA: effector memory T cell re-expressing CD45RA
THFA: tetrahydrofurfuryl alcohol
TNV: naïve T cell.

## References

[1] Lv JC Zhang LX. Prevalence and disease burden of chronic kidney disease. Adv Exp Med Biol. (2019) 1165:3–15. 10.1007/978-981-13-8871-2_1 31399958

[2] Thurlow JS Joshi M Yan G Norris KC Agodoa LY Yuan CM. Global epidemiology of end-stage kidney disease and disparities in kidney replacement therapy. Am J Nephrol. (2021) 52:98–107. 10.1159/000514550 33752206 PMC8057343

[3] Tonelli M Wiebe N Knoll G Bello A Browne S Jadhav D. Systematic review: kidney transplantation compared with dialysis in clinically relevant outcomes. Am J Transplant. (2011) 11:2093–109. 10.1111/j.1600-6143.2011.03686.x 21883901

[4] Hart A Smith JM Skeans MA Gustafson SK Wilk AR Castro S. OPTN/SRTR 2018 annual data report: kidney. Am J Transplant. (2020) 20:20–130. 10.1111/ajt.15672 31898417

[5] COPE. Official Journal of the international Society of Nephrology KDIGO 2012 Clinical Practice Guideline for the Evaluation and Management of Chronic Kidney Disease. Available online at: www.publicationethics.org (accessed April 23, 2022).

[6] Eikmans M Gielis EM Ledeganck KJ Yang J Abramowicz D Claas FFJ. Non-invasive biomarkers of acute rejection in kidney transplantation: novel targets and strategies. Front Med. (2019) 5:358. 10.3389/fmed.2018.00358 30671435 PMC6331461

[7] Van Loon E Naesens M. Blood transcriptomics as non-invasive marker for kidney transplant rejection. Nephrol Ther. (2021) 17S:S78–82. 10.1016/j.nephro.2020.02.012 33910703

[8] Guzzi F Cirillo L Buti E Becherucci F Errichiello C Roperto RM. Urinary biomarkers for diagnosis and prediction of acute kidney allograft rejection: a systematic review. Int J Mol Sci. (2020) 21:1–21. 10.3390/ijms21186889 32961825 PMC7555436

[9] Jin PH Sarwal RD Sarwal MM. Urinary biomarkers for kidney allograft injury. Transplantation. (2022) 106:1330–8. 10.1097/TP.0000000000004017 34982754

[10] Yang JYC Sarwal RD Sigdel TK Damm I Rosenbaum B Liberto JM. A urine score for non-invasive accurate diagnosis and prediction of kidney transplant rejection. Sci Transl Med. (2020) 12:eaba2501. 10.1126/scitranslmed.aba2501 32188722 PMC8289390

[11] Enghard P Rieder C Kopetschke K Klocke JR Undeutsch R Biesen R. Urinary CD4 T cells identify SLE patients with proliferative lupus nephritis and can be used to monitor treatment response. Ann Rheum Dis. (2014) 73:277–83. 10.1136/annrheumdis-2012-202784 23475982

[12] Bertolo M Baumgart S Durek P Peddinghaus A Mei H Rose T. Deep phenotyping of urinary leukocytes by mass cytometry reveals a leukocyte signature for early and non-invasive prediction of response to treatment in active lupus nephritis. Front Immunol. (2020) 11:256. 10.3389/fimmu.2020.00256 32265898 PMC7105605

[13] Roberti I Panico M Reisman L. Urine flow cytometry as a tool to differentiate acute allograft rejection from other causes of acute renal graft dysfunction. Transplantation. (1997) 64:731–4. 10.1097/00007890-199709150-00012 9311711

[14] Yu D-S Sun G-H Lee S-S Wu C-J Ma C-P Chang S-Y. Flow-Cytometric measurement of cellular changes in urine: a simple and rapid method for perioperatively monitoring patients after kidney transplantation. Urol Int. (1999) 62:143–6. 10.1159/000030378 10529663

[15] Roberti I Reisman L. Serial evaluation of cell surface markers for immune activation after acute renal allograft rejection by urine flow cytometry. Transplantation. (2001) 71:1317–20. 10.1097/00007890-200105150-00024 11397970

[16] Nanni-Costa A Iannelli S Vangelista A Buscaroli A Liviano G Raimondi C. Flow cytometry evaluation of urinary sediment in renal transplantation. Transpl Int. (1992) 5:S8–12. 10.1111/tri.1992.5.s1.8 14621719

[17] Galante NZ Câmara NOS Kallas EG Salomão R Pacheco-Silva A Medina-Pestana JO. Noninvasive immune monitoring assessed by flow cytometry and real time RT-PCR in urine of renal transplantation recipients. Transpl Immunol. (2006) 16:73–80. 10.1016/j.trim.2006.03.014 16860708

[18] van Doesum WB Abdulahad WH van Dijk MCRF Dolff S van Son WJ Stegeman CA. Characterization of urinary CD4+ and CD8+ T cells in kidney transplantation patients with polyomavirus BK infection and allograft rejection. Transpl Infect Dis. (2014) 16:733–43. 10.1111/tid.12273 25092256

[19] Nguan CYC Du C. Renal tubular epithelial cells as immunoregulatory cells in renal allograft rejection. Transplant Rev. (2009) 23:129–38. 10.1016/j.trre.2009.02.003 19361977

[20] Ting YT Coates PT Walker RJ McLellan AD. Urinary tubular biomarkers as potential early predictors of renal allograft rejection. Nephrology. (2012) 17:11–6. 10.1111/j.1440-1797.2011.01536.x 22050577

[21] Havasi A Dong Z. Autophagy and tubular cell death in the kidney. Semin Nephrol. (2016) 36:174. 10.1016/j.semnephrol.2016.03.005 27339383 PMC4920968

[22] Goerlich N Brand HA Langhans V Tesch S Schachtner T Koch B. Kidney transplant monitoring by urinary flow cytometry: biomarker combination of T cells, renal tubular epithelial cells, and podocalyxin-positive cells detects rejection. Sci Rep. (2020) 10:796. 10.1038/s41598-020-57524-7 31964937 PMC6972704

[23] Freund P Skopnik CM Metzke D Goerlich N Klocke J Grothgar E. Addition of formaldehyde releaser imidazolidinyl urea and MOPS buffer to urine samples enables delayed processing for flow cytometric analysis of urinary cells. medRxiv [Preprint]. (2022). 10.1101/2022.04.07.22273579 36880455

[24] Pradhan SK Guzman J Dargitz C Switalski S Landon M Olek S. Determination of immune cell identity and purity using epigenetic-based quantitative PCR. J Vis Exp. (2020) 2020:e60465. 10.3791/60465 32150154

[25] Baron U Werner J Schildknecht K Schulze JJ Mulu A Liebert UG. Epigenetic immune cell counting in human blood samples for immunodiagnostics. Sci Transl Med. (2018) 10:eaan3508. 10.1126/scitranslmed.aan3508 30068569

[26] R, Core Team,. R: The R Project for Statistical Computing. Available online at: https://www.r-project.org/ (accessed March 9, 2022).

[27] Paul RS Almokayad I Collins A Raj D Jagadeesan M. Donor-derived cell-free DNA: advancing a novel assay to new heights in renal transplantation. Transplant Direct. (2021) 7:3–438. 10.1097/TXD.0000000000001098 33564715 PMC7862009

[28] Burska AN Thu A Parmar R Bzoma I Samans B Raschke E. Quantifying circulating Th17 cells by qPCR: potential as diagnostic biomarker for rheumatoid arthritis. Rheumatology. (2019) 58:2015–24. 10.1093/rheumatology/kez162 31081041

[29] Le Cornet C Schildknecht K Chornet AR Fortner RT Maldonado SG Katzke VA. Circulating immune cell composition and cancer risk: a prospective study using epigenetic cell count measures. Cancer Res. (2020) 80:1885–92. 10.1158/0008-5472.CAN-19-3178 32075798

[30] Belmar Vega L Rodrigo Calabia E Gómez Román JJ Ruiz San Millán JC Martín Penagos L Arias Rodríguez M. Relationship between albuminuria during the first year and antibody-mediated rejection in protocol biopsies in kidney transplant recipients. Transplant Proc. (2016) 48:2950–2. 10.1016/j.transproceed.2016.09.012 27932115

[31] Oblak M Mlinšek G Kojc N Frelih M Buturović-Ponikvar J Arnol M. Spot urine protein excretion in the first year following kidney transplantation associates with allograft rejection phenotype at 1-year surveillance biopsies: an observational national-cohort study. Front Med. (2021) 8:781195. 10.3389/fmed.2021.781195 34869503 PMC8635090

[32] Hricik DE Nickerson P Formica RN Poggio ED Rush D Newell KA. Multicenter validation of urinary CXCL9 as a risk-stratifying biomarker for kidney transplant injury. Am J Transplant. (2013) 13:2634. 10.1111/ajt.12426 23968332 PMC3959786

[33] Hricik DE Augustine J Nickerson P Formica RN Poggio ED Rush D. Interferon gamma ELISPOT testing as a risk-stratifying biomarker for kidney transplant injury: results from the CTOT-01 multicenter study. Am J Transplant. (2015) 15:3166. 10.1111/ajt.13401 26226830 PMC4946339

