# Supplementary material for: Urinary CD8+HLA-DR+ T Cell Abundance Non-invasively Predicts Kidney Transplant Rejection
Source: Front Med (Lausanne). 2022 Jul 15;9:928516. doi: 10.3389/fmed.2022.928516 (PMC9334669; doi:10.3389/fmed.2022.928516)
Supplement: Supplementary file 1 [file Data_Sheet_1.docx]

Supplementary Material

# Supplementary Data

Supplementary Material should be uploaded separately on submission. Please include any supplementary data, figures and/or tables. All supplementary files are deposited to FigShare for permanent storage and receive a DOI.

Supplementary material is not typeset so please ensure that all information is clearly presented, the appropriate caption is included in the file and not in the manuscript, and that the style conforms to the rest of the article. To avoid discrepancies between the published article and the supplementary material, please do not add the title, author list, affiliations or correspondence in the supplementary files.

# Supplementary Figures and Tables

For more information on Supplementary Material and for details on the different file types accepted, please see [here](http://home.frontiersin.org/about/author-guidelines#SupplementaryMaterial). Figures, tables, and images will be published under a Creative Commons CC-BY licence and permission must be obtained for use of copyrighted material from other sources (including re-published/adapted/modified/partial figures and images from the internet). It is the responsibility of the authors to acquire the licenses, to follow any citation instructions requested by third-party rights holders, and cover any supplementary charges.


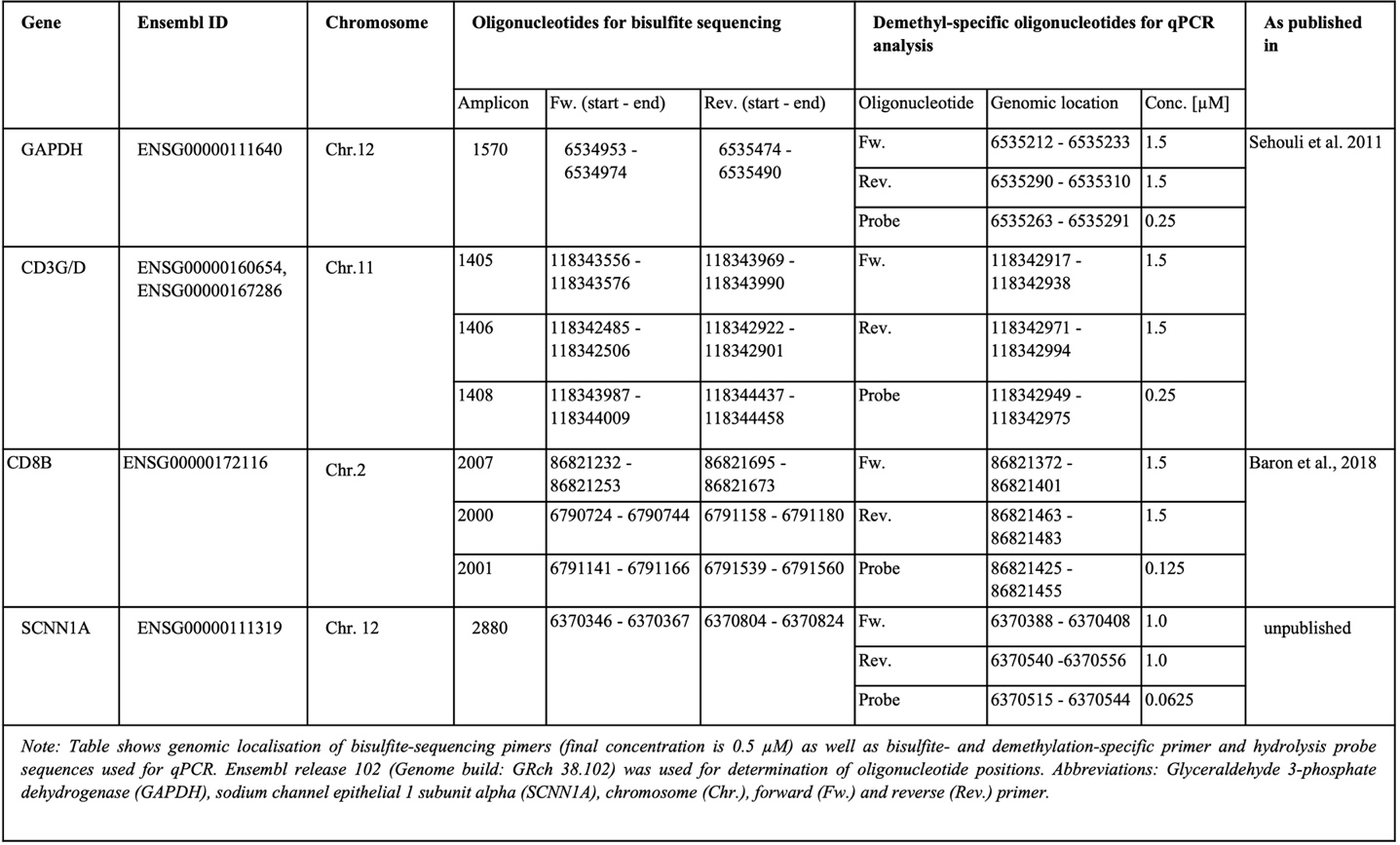


| **Gene** | **Ensembl ID** | **Chromosome** | **Oligonucleotides for bisulfite sequencing** | | | **Demethyl-specific oligonucleotides for qPCR analysis** | | | **As published in** |
| --- | --- | --- | --- | --- | --- | --- | --- | --- | --- |
|  |  |  | Amplicon | Fw. (start - end) | Rev. (start - end) | Oligonucleotide | Genomic location | Conc. [µM] |  |
| GAPDH | ENSG00000111640 | Chr.12 | 1570 | 6534953 - 6534974 | 6535474 - 6535490 | Fw. | 6535212 - 6535233 | 1.5 | Sehouli et al. 2011 |
|  |  |  |  |  |  | Rev. | 6535290 - 6535310 | 1.5 |  |
|  |  |  |  |  |  | Probe | 6535263 - 6535291 | 0.25 |  |
| CD3G/D | ENSG00000160654, ENSG00000167286 | Chr.11 | 1405 | 118343556 - 118343576 | 118343969 - 118343990 | Fw. | 118342917 - 118342938 | 1.5 |  |
|  |  |  | 1406 | 118342485 - 118342506 | 118342922 - 118342901 | Rev. | 118342971 - 118342994 | 1.5 |  |
|  |  |  | 1408 | 118343987 - 118344009 | 118344437 - 118344458 | Probe | 118342949 - 118342975 | 0.25 |  |
| CD8B | ENSG00000172116 | Chr.2 | 2007 | 86821232 - 86821253 | 86821695 - 86821673 | Fw. | 86821372 - 86821401 | 1.5 | Baron et al., 2018 |
|  |  |  | 2000 | 6790724 - 6790744 | 6791158 - 6791180 | Rev. | 86821463 - 86821483 | 1.5 |  |
|  |  |  | 2001 | 6791141 - 6791166 | 6791539 - 6791560 | Probe | 86821425 - 86821455 | 0.125 |  |
| SCNN1A | ENSG00000111319 | Chr. 12 | 2880 | 6370346 - 6370367 | 6370804 - 6370824 | Fw. | 6370388 - 6370408 | 1.0 | unpublished |
|  |  |  |  |  |  | Rev. | 6370540 -6370556 | 1.0 |  |
|  |  |  |  |  |  | Probe | 6370515 - 6370544 | 0.0625 |  |
| *Note: Table shows genomic localisation of bisulfite-sequencing pimers (final concentration is 0.5 µM) as well as bisulfite- and demethylation-specific primer and hydrolysis probe sequences used for qPCR. Ensembl release 102 (Genome build: GRch 38.102) was used for determination of oligonucleotide positions. Abbreviations: Glyceraldehyde 3-phosphate dehydrogenase (GAPDH), sodium channel epithelial 1 subunit alpha (SCNN1A), chromosome (Chr.), forward (Fw.) and reverse (Rev.) primer.* | | | | | | | | | |

**Supplementary Table 1.** Table shows genomic localisation of bisulfite-sequencing primers (final concentration is 0.5 µM) as well as bisulfite- and demethylation-specific primer and hydrolysis probe sequences used for qPCR. Ensembl release 102 (Genome build: GRch 38.102) was used for determination of oligonucleotide positions. GAPDH, Glyceraldehyde 3-phosphate dehydrogenase; SCNN1A, sodium channel epithelial 1 subunit alpha; Chr., chromosome; Fw., forward primer; Rev., reverse primer.

**Supplementary Figure 1.** Methylation matrix of the SCNN1A-specific marker. The matrix illustrates the differentially methylated region within the sodium channel epithelial 1 subunit alpha (SCNN1A) gene based on bisulfite-sequencing (amplicon 2880 (AMP2880)). This region was identified as a potential marker specific for proximal tubular epithelial cells (PTEC; red letters). Different cell populations were analysed (y-axis). CpG-Dinukleotide (relative CpG-No. 1-8 on x-axis) of AMP2880 are illustrated as boxes. The methylation value of each CpG is color-coded (100% methylated: blue, 0% methylated: yellow). These values were determined by bisulfite-sequencing as described previously (Lewin et al., Bioinformatics. 2004). Only CpG-positions 4-8 were targeted by the qPCR assay (CpG-No.: 7-8 (forward primer), 4 (reverse primer) and 4 5 (probe)). vWF, von Willebrand factor; PECAM, platelet endothelial cell adhesion molecule.


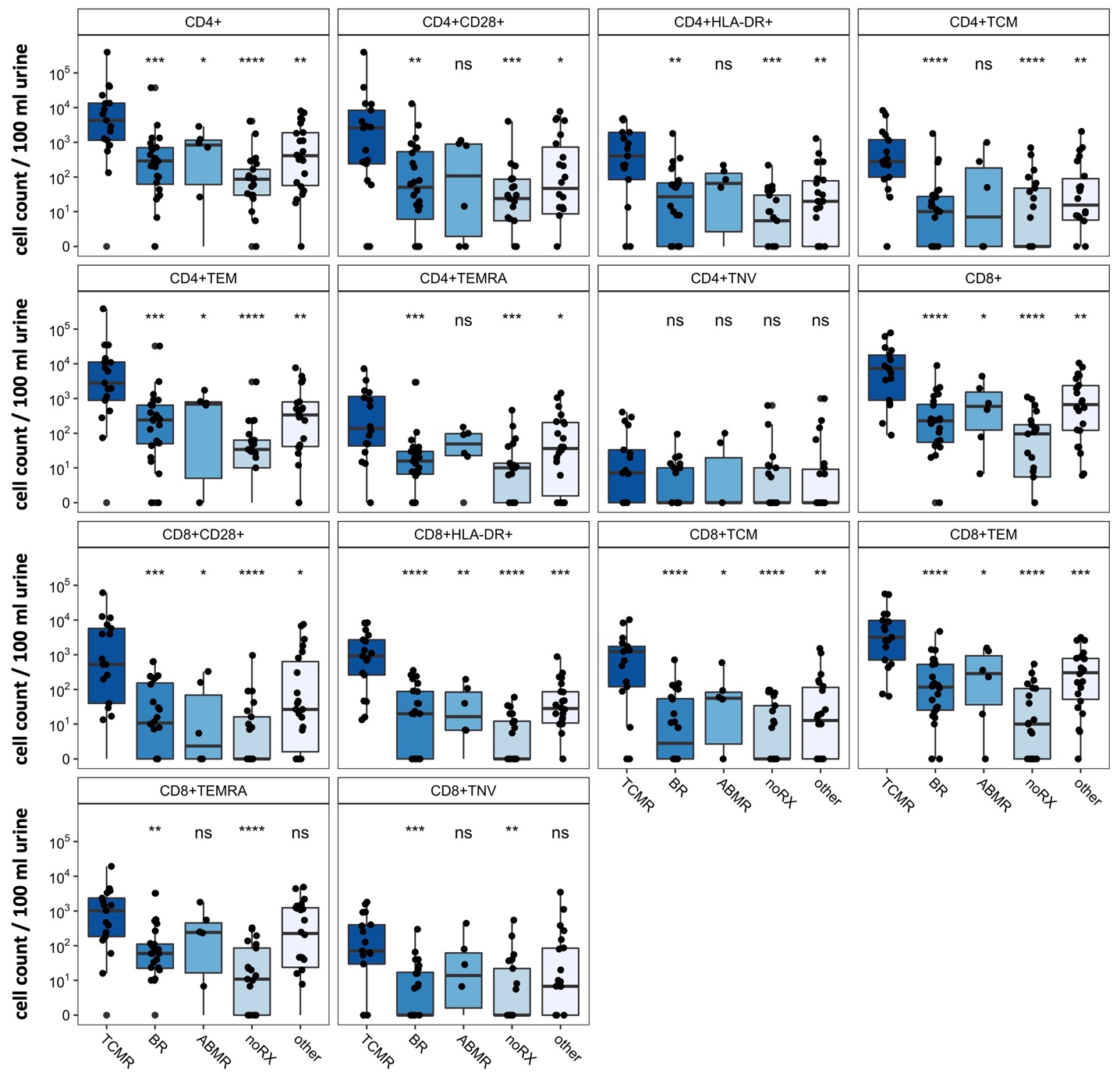


**Supplementary Figure 2.** Overview of T cell subsets investigated in this study. Patients are subdivided into five groups based on histopathological results from biopsy. T cell counts per 100 ml urine shown for different biopsy groups of KT patients with graft deterioration. ns, no significance; *p < 0.05; **p < 0.01; ***p < 0.001; ****p < 0.0001. TCMR, T cell-mediated rejection; BR, Borderline rejection; ABMR, antibody-mediated rejection; noRX, no rejection; other, other pathologies; TEC, tubular epithelial cell.

**
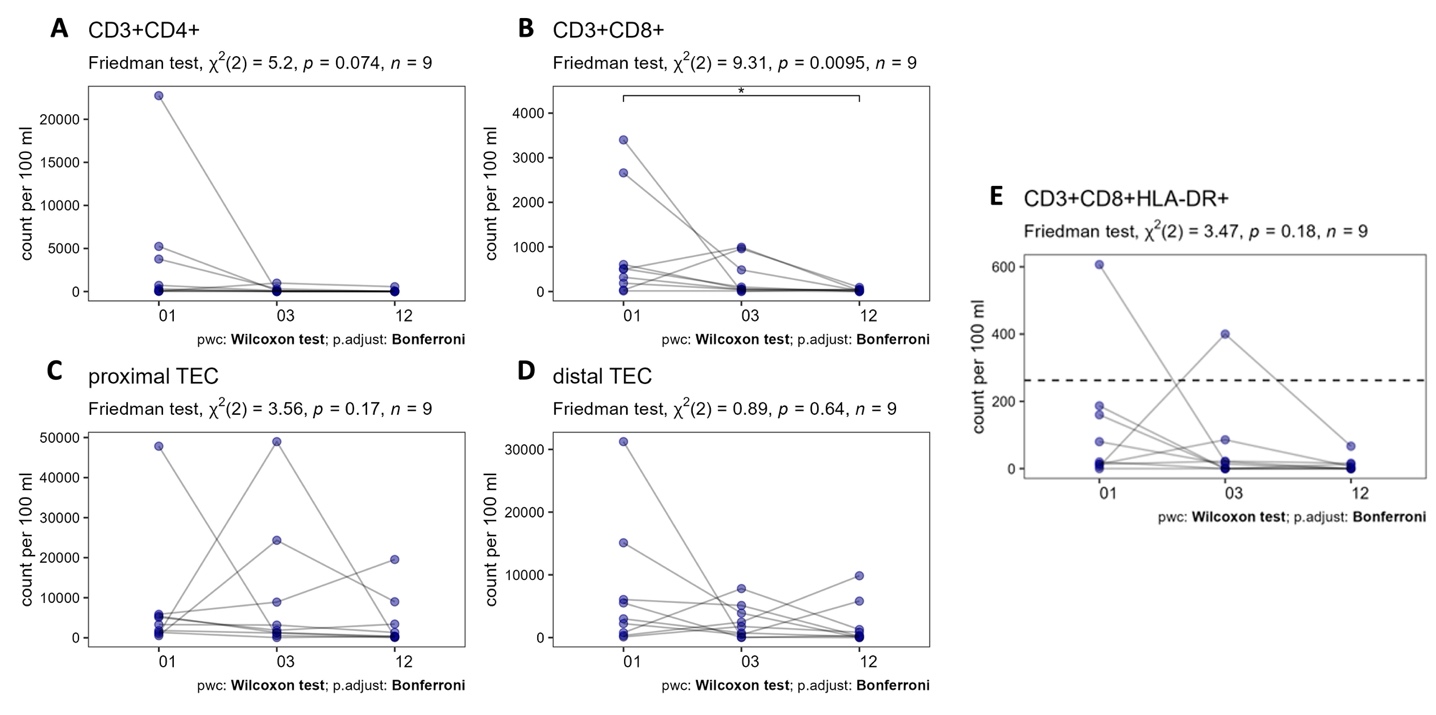
**

**Supplementary Figure 3.** Urinary T cell and TEC subset abundance over time in 9 patients measured at three timepoints (one, three and 12 months) after transplantation. Single measurements are displayed as points. Intraindividual trajectories are displayed by lines. Dashed line indicates the urinary CD8+HLA-DR+ biomarker cut-off. *p < 0.05, ns, no significance; TEC, tubular epithelial cell.
